# Sex tourism among Chinese men who have sex with men: a cross-sectional observational study

**DOI:** 10.1186/s12889-018-5214-2

**Published:** 2018-03-02

**Authors:** Jessica Mao, Weiming Tang, Chuncheng Liu, Ngai Sze Wong, Songyuan Tang, Chongyi Wei, Joseph D. Tucker

**Affiliations:** 1 University of North Carolina Project China, 2 Lujing Road, Floor 11., Guangzhou, Guangdong 510000 China; 2 The Johns Hopkins Hospital, Baltimore, USA; 3 University of North Carolina Chapel Hill, Chapel Hill, USA; 4 Rutgers School of Public Health, New Brunswick, USA; 5 Faculty of Infectious and Tropical Diseases, London School of Hygiene and Tropical Medicine, London, UK

**Keywords:** Men who have sex with men (MSM), China, HIV/AIDS, Sexually transmitted diseases, Migration

## Abstract

**Background:**

Sex tourism among men who have sex with men (MSM) may exacerbate transmission of HIV and other sexually transmitted infections (STIs). Sex tourism is defined as purchasing sex with gifts or money outside of one’s hometown. Our objective was to characterize the frequency, socio-demographic characteristics, and sexual risk behaviors among Chinese MSM sex tourists.

**Methods:**

An online, cross-sectional survey for high-risk MSM throughout China was conducted in November 2015 covering sociodemographic characteristics, sexual risk behaviors, and sex tourism. Univariate and multivariable logistic regressions were performed to identify correlates of sex tourism. The mean MSM HIV prevalence of sex tourism journey origins and destinations were compared.

**Results:**

Of 1189 MSM who completed the survey, 62 (5%) men identified as sex tourists; among these sex tourists, twenty (32%) traveled primarily to purchase sex and the remainder purchased sex while traveling for another purpose. There was minimal socio-demographic and behavioral difference between the two groups. In multivariable analyses, adjusting for age and income, sex tourism was correlated with high-risk sexual behaviors, higher income (aOR 4.44, 95%CI 1.77–11.18) and living with HIV (aOR 2.79, 95%CI 1.03–7.55). Sex tourism was more often from locations with lower to higher MSM HIV prevalence (mean = 4.47, SD = 2.01 versus mean = 6.86, SD = 5.24).

**Conclusion:**

MSM sex tourists were more likely to have risky sexual behaviors and travel to locations with a higher HIV prevalence. MSM sex tourists may be part of core groups that are disproportionately responsible for MSM HIV transmission. Enhanced surveillance and interventions tailored to MSM sex tourists should be considered.

## Background

Local stigma associated with men who have sex with men (MSM) in low and middle-income countries is widespread. [1] Formal and informal condemnation of MSM in these settings encourage many men to hide their sexual orientation, especially in their hometowns. [2] This social pressure may contribute to an increase in sex tourism. [3] In this study, we define sex tourism as having traveled outside of one’s hometown to purchase sex in exchange for money or gifts.

Sex tourism may be important for HIV control for three reasons. First, this human movement could connect areas of low HIV prevalence and areas of high HIV prevalence. [4] The movement of high-risk individuals across areas with markedly different HIV prevalence has been shown to drive HIV transmission. [5, 6] Second, as sex tourists are a subgroup of clients of male sex workers, they often have greater power in deciding about condom use. [6] Third, traveling for sex is linked with unprotected anal intercourse and other behaviors that increase HIV risk. [7–10]

While the literature on sex tourism has traditionally focused on men traveling in search of women, the topic of MSM sex tourism has recently become more common. [11] In addition, sex tourists were previously more common from high-income countries traveling to lower-income countries, but sex tourism originating from low and middle-income countries is increasingly common. China presents a strong location to study sex tourism among online MSM because of a growing number of domestic and international tourists [12] and a culturally repressed MSM society that is subject to numerous societal pressures to stay closeted. [4]

The purpose of this study was to characterize sex tourism in the context of Chinese MSM at high risk for HIV infection. We conducted an online survey to determine socio-demographic and behavioral correlates of MSM sex tourism in China.

## Methods

### Participant recruitment and survey development

In November 2015, MSM in China were recruited to participate in a nationwide online, cross-sectional survey. The survey was anonymous and voluntary. Survey promotion took place online primarily through Danlan.org, an online gay portal, and its gay mobile dating app BlueD. A number of men were also recruited through social media platforms such as Weibo, a microblogging platform and WeChat, a messaging app. The banner ad asked for men who had ever had sex with men to complete a brief survey in return for compensation. Upon clicking the ad, men were directed to the survey, hosted on Qualtrics (Provo, Utah). Men were excluded if they were under 16 years of age, not born biologically male, had never had anal sex with a man, and had not had condomless sex within the last three months. Men who met eligibility criteria provided informed consent. For this study, we defined high-risk individuals as those who had participated in condomless sex within the last three months. Those who did not meet inclusion criteria were excluded using the above criteria. The survey was run until the sample size of 1170 participants was met, approximately 4 days. All promotional materials and the survey itself were written in the Chinese language.

A more detailed description of survey preparation and participant recruitment is reported elsewhere. [13] Sex tourism questions were developed after a review of current literature and field tested with 150 MSM. The CONSORT E-health checklist [14] for online surveys was used to ensure completeness.

### Measures

Sociodemographic and sex behavior survey items were used in a previous online MSM research survey. [15] Survey questions collected data on sociodemographic information, including age, residence, marital status, education, and income. Participants were also asked about their self-identified sexual orientation, whether or not they had ever disclosed their orientation to a healthcare worker, number of male and/or female sex partners in the last three months, whether sex partners were primary or casual partners, and condom use behaviors. Questions also covered sexual risk behaviors, such as sex while drunk, sex under the influence of drugs, group sex, and paid sex.

In our survey, sex tourism was defined as having traveled outside of the city of residence to purchase sex with gifts or money. Participants answering that they had participated in sex tourism were then asked whether they traveled for the primary purpose of purchasing sex (primary sex tourists) or for another primary reason (non-primary sex tourists), where they traveled, how they found sex partners at their destination, gender and ethnicity of partner from whom they purchased services, reasons for participating in sex tourism, condom use behaviors while traveling, price paid, whether they traveled alone and whether they had discussed HIV status prior to intercourse.

### Statistical analysis

Descriptive analysis, including frequency of observed behavior, was completed comparing the sex tourists to the general survey population. Pearson’s chi-squared test was applied to determine the independence of observed variables to outcomes. Univariate analysis was completed to identify which variables were correlated with having participated in sex tourism. Multivariable logistic regression analyses were performed adjusting for age and income. All statistical analyses were completed using SAS (Cary, NC).

The prevalence of HIV among MSM at each sex tourist’s origin hometown and the destination was obtained from existing literature. [16–21] All Chinese and international prevalence data were taken from the most recent published source. Analysis of MSM HIV prevalence among was completed by comparing the MSM HIV prevalence at the origin and destination of each journey made by a sex tourist. The HIV prevalence among MSM was compared between each sex tourist’s home province in China and destination province in China (if a domestic journey) or destination country (if an international journey). The average HIV prevalence among MSM at the journey origins and destinations were compared using a paired T-test.

### Ethics

Ethical approval was obtained from institutional review boards at the Guangdong Provincial Center for Skin Diseases and Sexually Transmitted Infections Control (IRB number 1R01AI114310–01), the University of North Carolina at Chapel Hill (IRB number 14–1865), and the University of California San Francisco (IRB number 14–14887). All participants signed an online informed consent form prior to beginning the survey detailing what information would be collected and what the data would be used for. Those who refused to sign the informed consent were not allowed to proceed with the survey.

## Results

In total, 7892 people clicked on the banner link to enter the survey, and 7551 (96%) started the survey. Of the 1597 participants who passed the exclusion criteria and agreed to the online informed consent, 1189 participants completed the survey. The majority of study participants were under 30 years old (82%), had never been married (83%), identified as gay (70%), had been educated beyond high school (68%), with an average income of less than $10,000 United States Dollars (USD) annually (82%). Full statistics of the study population are reported in Table 1.

**Table 1:** Socio-demographic and behavioral data of high-risk MSM in China in 2015 (*N* = 1189)

Demographic or Behavior	(+) Sex Tourism (*N* = 62)	(−) Sex Tourism (*N* = 1127)	Overall Total (N = 1189)	Χ^2^ (p)
	N (%)	N (%)	N (%)	
Age (years)				11.38 (*p* = 0.01)
16–20	10 (16)	313 (28)	323 (27)	
21–25	18 (50)	409 (36)	427 (36)	
26–30	13 (21)	211 (19)	224 (19)	
> 30	20 (34)	195 (17)	215 (18)	
Residence (Region)				3.34 (*p* = 0.65)
Northern China	15 (24)	236 (21)	251 (21)	
Northeastern China	8 (13)	89 (8)	97 (8)	
Eastern China	17 (27)	311 (28)	328 (28)	
Southern China	14 (23)	278 (25)	292 (25)	
Southwestern China	5 (8)	131 (12)	136 (11)	
Northwestern China	3 (5)	82 (7)	85 (7)	
Marital status				2.68 (*p* = 0.10)
Single	47 (76)	944 (84)	991 (83)	
Ever married	15 (24)	183 (16)	198 (17)	
Education level				22.34 (*p* < 0.001)
High school or less	9 (15)	383 (34)	392 (33)	
College	44 (71)	700 (62)	744 (63)	
Post-graduate	9 (15)	44 (4)	53 (5)	
Annual Income (USD)				45.97 (*p* < 0.001)
< 3000	8 (13)	322 (29)	330 (28)	
3000–10,000	23 (37)	622 (55)	645 (54)	
10,000–15,000	20 (32)	119 (11)	139 (12)	
> 15,000	11 (18)	64 (6)	75 (6)	
Self-Identified Sexual Identity				21.96 (*p* < 0.001)
Homosexual	43 (69)	792 (70)	835 (70)	
Bisexual	13 (21)	295 (26)	308 (26)	
Heterosexual	1 (2)	0 (0)	1 (0)	
Other	5 (8)	40 (4)	45 (4)	
“Out” to doctor ^a^				4.78 (*p* = 0.03)
Yes	27 (44)	342 (30)	369 (31)	
No	35 (56)	785 (70)	820 (69)	
Primary male partner				0.003 (*p* = 0.95)
Yes	44 (75)	796 (71)	840 (71)	
No	18 (25)	331 (29)	349 (29)	
Primary female partner				2.40 (*p* = 0.12)
Yes	11 (18)	127 (11)	138 (12)	
No	51 (82)	1000 (89)	1051 (88)	
> 1 sex partner in last 3 months				6.28 (*p* = 0.01)
Yes	43 (69)	598 (53)	641 (54)	
No	19 (31)	529 (47)	548 (46)	
Number of Partners				13.17 (*p* = 0.004)
1	19 (31)	527 (47)	546 (46)	
2–3	24 (39)	435 (39)	459 (39)	
4–5	12 (19)	109 (10)	121 (10)	
6+	7 (11)	56 (5)	63 (5)	
Condomless sex in last month^b^				6.0 (p = 0.01)
Yes	23 (37)	264 (23)	287 (24)	
No	39 (63)	863 (76)	902 (76)	
Condomless sex with male in last month				3.20 (*p* = 0.07)
Yes	20 (32)	253 (22)	273 (23)	
No	42 (68)	874 (78)	916 (77)	
Condomless sex with female in last month				9.27 (*p* = 0.002)
Yes	5 (8)	23 (2)	28 (2)	
No	57 (92)	1104 (98)	1161 (98)	
Sex while drunk				38.72 (*p* < 0.001)
Yes	25 (40)	139 (12)	164 (14)	
No	37 (60)	988 (88)	1025 (86)	
Group Sex ^c^				9.77 (*p* = 0.002)
Yes	13 (21)	101 (9)	114 (10)	
No	49 (79)	1026 (91)	1075 (90)	
Been paid for sex				7.64 (*p* = 0.005)
Yes	16 (26)	150 (13)	166 (14)	
No	46 (74)	977 (87)	1023 (86)	
Ever HIV tested				5.89 (*p* = 0.02)
Yes	43 (69)	604 (54)	647 (54)	
No	19 (31)	523 (46)	542 (46)	
Ever received HIV positive result				4.45 (p = 0.03)
Yes	5 (8)	35 (3)	40 (3)	
No	57 (92)	905 (97)	953 (97)	
Ever tested for syphilis				9.04 (p = 0.002)
Yes	29 (47)	325 (29)	354 (30)	
No	33 (53)	802 (71)	835 (70)	
Ever received STD diagnosis or treatment				0.31 (*p* = 0.58)
Yes	14 (23)	222 (20)	236 (20)	
No	48 (77)	905 (80)	953 (80)	

^a “^Out to a doctor” was defined as having ever discussed sexual orientation with a healthcare provider; ^b^ Condomless sex is defined as any incidence of sexual intercourse without use of a condom; ^c^ Group sex was defined as any incidence of sexual intercourse involving more than two people

### Characteristics of sex tourism

Sixty-two of the 1189 participants (5%) reported sex tourism, and of these 62 men, 20 (32%) traveled for the primary purpose of purchasing sex. Forty-two (68%) men reported that they purchased sex while traveling for another primary reason. Of the 62 sex tourists, 76% purchased sexual services from two or fewer partners, and 87% purchased services from male partners. Of the 92 journeys made by the 62 sex tourists, 75% of them were domestic, with Beijing as the most popular destination, followed by Shanghai and Guangzhou. Thailand was the most popular international destination, followed by Japan and Russia. The primary reasons for participating in sex tourism were desire to try sex with a man (34%) and fear of recognition at home (29%). The frequency of condomless sex while sex touring (33%) was comparable to the rate of condomless sex in the most recent month prior to taking the survey (37%). More details regarding characteristics of sex tourists can be found in Table 2.

**Table 2:** Sex tourism among high-risk MSM in China, 2015 (N = 62)

Variable	Prevalence
	*N* (%)
Primary purpose of travel to purchase sex	
Yes	20 (32)
No	42 (68)
Destination	
Within China	69 (75)
Outside China	23 (25)
How was partner found	
Mobile App	39 (63)
Website/Online portal	29 (47)
In-person solicitation	12 (19)
Local Establishment	11 (18)
Average distance traveled to purchase sex	
Within China	1818.7 km
< 500 km	43 (80)
501–1000 km	8 (16)
1001–2000 km	18 (33)
> 2000 km	8 (15)
Outside China	4316.0 km
< 2500 km	6 (43)
2501–5000 km	5 (36)
> 5000 km	5 (36)
Ethnicity of Partner from whom services were purchased	
Native to destination country	59 (95)
Not native to destination country	3 (5)
Reason for sex tourism	
Wanted to try sex with a man	21 (34)
Afraid of recognition at home	18 (29)
Price	7 (11)
Do not need to use condoms	2 (3)
Unable to get at home	11 (18)
Drunk	4 (7)
Other	17 (27)
Purchased sex from	
Women	8 (13)
Men	54 (87)
Transgender	2 (3)
Number of partners purchased while traveling	
1	35 (56)
2–5	12 (20)
3–5	9 (15)
6–10	2 (3)
> 10	3 (3)
Condomless sex	
Yes, vaginal	3 (5)
Yes, anal	20 (32)
No	41 (66)
Reason for condomless sex	
I did not want to use	17 (27)
Did not have time	8 (13)
My partner did not want to use	6 (10)
Did not have one	4 (7)
I believed I was HIV negative	4 (7)
I believed my partner was HIV negative	4 (7)
Drunk or high	1 (2)
Price paid (USD)	
< 75	39 (63)
75–150	14 (23)
150–300	5 (8)
> 300	3 (5)
Travel Alone?	
Yes	41 (66)
No	21 (34)
Did you ask partner about HIV status?	
Yes	24 (39)
No	37 (61)
Have you ever tested for HIV/frequency	
No	20 (32)
Less than every 2 years	6 (10)
Once a year	19 (31)
Once every 6 months	12 (19)
Once every 3 months	5 (8)
Have you ever tested for syphilis?	
Yes	28 (45)
No	34 (55)

In comparing the two subsets of primary sex tourists and non-primary sex tourists, we found that the two groups were comparable in terms of age, education, income, self-identified sexual orientation, the disclosure of orientation to a healthcare provider, and number of sex partners. Among the primary sex tourists, we observed a higher frequency of international travel (36% versus 18%), a higher average price paid for services ($200 USD versus $85 USD), a greater frequency of condomless sex while traveling (70% versus 21%). These differences proved to be purely observational with chi-squared tests showing no significant correlation of behaviors to whether individuals were primary or non-primary sex tourists. A detailed comparison of the two groups can be found in Table 3.

**Table 3:** Comparison of socio-demographic and behavioral data between primary sex tourists^a^ (*n* = 20) and non-primary sex tourists^b^ (*n* = 42)

Demographic or Behavior	Primary Sex Tourism (*N* = 20)	Not Primary Sex Tourism (*N* = 42)	Overall Total (*N* = 62)	Χ^2^ (p)
	N (%)	N (%)	N (%)	
Age (years)				0.45 (*p* = 0.92)
16–20	3 (15)	7 (17)	10 (16)	
21–25	5 (25)	13 (32)	18 (50)	
26–30	5 (25)	8 (20)	13 (21)	
> 30	7 (35)	13 (32)	20 (34)	
Residence (Region)				4.75 (*p* = 0.45)
Northern China	5 (25)	10 (16)	15 (24)	
Northeastern China	0 (0)	8 (13)	8 (13)	
Eastern China	6 (30)	11 (18)	17 (27)	
Southern China	6 (30)	8 (13)	14 (23)	
Southwestern China	2 (10)	3 (5)	5 (8)	
Northwestern China	1 (5)	2 (3)	3 (5)	
Marital status				3.24 (*p* = 0.07)
Single	18 (90)	29 (69)	47 (76)	
Ever married	2 (10)	13 (31)	15 (24)	
Education level				1.03 (*p* = 0.60)
High school or less	2 (10)	7 (17)	9 (15)	
College	14 (70)	30 (71)	44 (71)	
Post-graduate	4 (20)	5 (12)	9 (15)	
Annual Income (USD)				3.82 (*p* = 0.28)
< 3000	1 (5)	7 (17)	8 (13)	
3000–10,000	10 (50)	13 (31)	23 (37)	
10,000–15,000	7 (35)	13 (31)	20 (32)	
> 15,000	2 (10)	9 (21)	11 (18)	
Self-Identified Sexual Orientation				2.33 (*p* = 0.51)
Gay	13 (65)	30 (71)	43 (69)	
Bisexual	4 (20)	9 (21)	13 (21)	
Straight	1 (5)	0 (0)	1 (2)	
Other	2 (10)	3 (7)	5 (8)	
“Out” to doctor^c^				0.03 (*p* = 0.87)
Yes	9 (45)	18 (43)	27 (44)	
No	11 (55)	24 (57)	35 (56)	
Primary male partner				0.51 (*p* = 0.47)
Yes	13 (65)	31 (74)	44 (75)	
No	7 (35)	11 (26)	18 (25)	
Primary female partner				0.15 (*p* = 0.70)
Yes	3 (15)	8 (19)	11 (18)	
No	17 (85)	34 (81)	51 (82)	
> 1 sex partner in last 3 months				0.85 (*p* = 0.35)
Yes	13 (65)	32 (76)	45 (73)	
No	7 (35)	10 (24)	17 (27)	
Number of Partners				1.97 (*p* = 0.58)
1	7 (35)	10 (24)	19 (31)	
2–3	8 (40)	16 (38)	24 (39)	
4–5	2 (10)	10 (24)	12 (19)	
6+	3 (15)	6 (14)	7 (11)	
Condomless sex in last month ^d^				0.30 (*p* = 0.86)
Yes, with man	7 (35)	13 (31)	20 (32)	
Yes, with woman	2 (10)	3 (7)	5 (8)	
No	12 (60)	28 (67)	37 (60)	
Sex while drunk				1.63 (*p* = 0.20)
Yes	11 (55)	30 (71)	25 (40)	
No	9 (45)	12 (29)	37 (60)	
Group Sex^e^				3.75 (*p* = 0.05)
Yes	7 (35)	6 (14)	13 (21)	
No	13 (65)	36 (86)	49 (79)	
Been paid for sex				3.11 (*p* = 0.08)
Yes	8 (40)	8 (19)	16 (26)	
No	12 (60)	34 (81)	46 (74)	
Ever HIV tested				0.10 (*p* = 0.75)
Yes	13 (65)	29 (69)	42 (68)	
No	7 (35)	13 (31)	20 (32)	
Ever tested for syphilis				2.74 (*p* = 0.10)
Yes	6 (30)	22 (52)	29 (47)	
No	14 (70)	20 (48)	33 (53)	
Ever received STD diagnosis or treatment				0.29 (*p* = 0.59)
Yes	5 (25)	8 (19)	13 (21)	
No	15 (75)	34 (81)	49 (79)	

^a^Primary sex tourists defined as those who traveled for the primary purpose of purchasing sex, ^b^Non-primary sex tourists are defined as those who purchased sex while traveling for other reasons. ^c “^Out to a doctor” was defined as having ever discussed sexual orientation with a healthcare provider; ^d^ Condomless sex is defined as any incidence of sexual intercourse without use of a condom; ^e^ Group sex was defined as any incidence of sexual intercourse involving more than two people

### Differences in sociodemographic and behavioral characteristics between sex tourists and non-sex tourists

In comparing the 62 sex tourists to non-sex tourists, sex tourists tended to be older (64% of sex tourists were under 30 years old vs. 83% of non-sex tourists), married (76% single vs. 84% single), and more educated (85% educated beyond high school vs. 66%). In looking at how many sex partners participants had in the last three months, sex tourists were more likely to have had more than three sex partners (31% vs. 15%), and were more likely to have participated in condomless sex in the last month (37% vs. 24%).

There was a higher frequency of HIV testing among sex tourists (69% of sex tourists compared to 54% in non-sex tourists) and more sex tourists had received a positive HIV result compared to non-sex tourists (9% compared to 3%) (Table 1).

Univariate and multivariable analyses were performed to identify correlates of sex tourism. Multivariable analyses were adjusted for age and income. Age was chosen as many other variables, including education level, marital status, income, lifetime number of partners, and having ever participated in other risk activities or healthcare screening were thought to all be likely affected by age. Income was chosen as an adjustment variable as major factors of our study, including travel and payment for services, are more feasible for those with expendable income. Higher income (adjusted only for age, adjusted odds ratio (aOR) 4.44, 95%CI 1.77–11.18) and higher education were correlated with sex tourism (aOR 2.26, 95%CI 1.29–5.54 and 8.71, 95%CI 3.28–32.08 for college and graduate school, respectively). The likelihood of participating in sex tourism was higher among those reporting more than 6 sex partners in the last three months (aOR 2.94, 95%CI 1.15–7.53), sex while drunk (aOR 4.67, 95%CI 2.63–8.27), group sex (aOR 2.81, 95%CI 1.44–5.51), and having ever been paid for sex (aOR 3.18, 95%CI 1.68–6.02). HIV testing and syphilis testing were both positively correlated with sex tourism in univariate analysis (crude odds ratio (cOR) 1.96, 95%CI 1.13–3.41 and cOR 2.21, 95%CI 1.29–3.63, respectively) but this correlation disappeared after adjusting for age, income and education. Detailed results from univariate and multivariate analyses are reported in Table 4**.**

**Table 4:** Correlates of men having participated in sex tourism among MSM in China (n = 62) when compared to men who did not (n = 1127)

Variable	Crude OR (95% CI)	Adjusted OR (95% CI) ^a^
Age ^b^	1.06 (1.03–1.09)	1.03 (0.99–1.07)
Education level		
High school or less	Ref	Ref ^c^
College or higher	2.68 (1.29–5.54)	2.26 (1.08–4.72)
Post-graduate	8.71 (3.28–23.08)	5.19 (1.87–14.38)
Marital status		
Single	0.61 (0.33–1.11)	0.47 (0.35–1.62)
Ever Married	Ref	Ref
Annual Income (USD)		
< 3000	Ref	Ref ^d^
3000–10,000	1.49 (0.66–3.37)	1.23 (0.52–2.90)
10,000–15,000	6.77 (2.90–15.77)	5.21 (2.09–13.01)
> 15,000	6.92 (2.68–17.88)	4.41 (1.51–12.86)
Self-identified sexual orientation		
Gay	0.96 (0.55–1.67)	1.08 (0.61–1.91)
Bisexual / Straight / Other	Ref	Ref
“Out” to healthcare worker		
Yes	Ref	Ref
No	0.57 (0.34–0.95)	0.61 (0.36–1.03)
Primary Male partner		
Yes	1.08 (0.59–1.97)	1.01 (0.54–1.89)
No	Ref	Ref
Primary Female partner		
Yes, female	0.39 (0.11–1.36)	0.31 (0.08–1.15)
No	Ref	Ref
Number of sexual partners in last 3 months		
1	Ref	Ref
2–3	1.53 (0.83–2.83)	1.28 (0.68–2.41)
4–5	3.81 (1.53–9.49)	2.58 (0.99–6.67)
6+	2.92 (1.38–6.18)	2.29 (1.06–4.97)
Condomless Sex		
Yes	1.93 (1.13–3.29)	1.83 (1.06–3.17)
No	Ref	Ref
Sex while drunk		
Yes	4.80 (2.81–8.22)	4.24 (2.44–7.40)
No	Ref	Ref
Have participated in group sex		
Yes	2.70 (1.41–5.14)	2.81 (1.44–5.51)
No	Ref	Ref
Been paid for sex		
Yes	2.27 (1.25–4.11)	2.81 (1.52–5.22)
No	Ref	Ref
Ever HIV tested		
Yes	1.96 (1.13–3.41)	1.50 (0.85–2.65)
No	Ref	Ref
Ever received HIV positive result		
Yes	2.74 (1.03–7.25)	2.79 (1.03–7.55)
No	Ref	Ref
Have tested for syphilis		
Yes	2.17 (1.29–3.63)	1.62 (0.95–2.77)
No	Ref	Ref
Ever received STD treatment or diagnosis		
Yes	1.19 (0.64–2.20)	1.09 (0.58–2.07)
No	Ref	Ref

^a^Multivariable analysis controlled for age and income unless otherwise specified; ^b^ Age was analyzed as a continuous variable; ^c^ Multivariable analysis of education level controlled for age and income; ^d^ Multivariable analysis of income controlled for age and education

### Geographical HIV prevalence

**Fig. 1:**
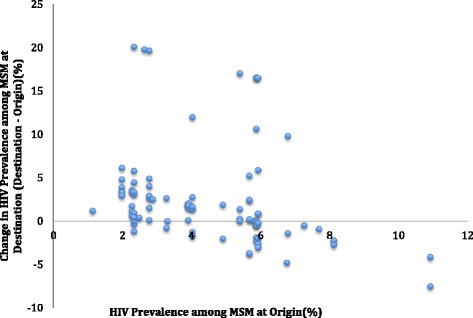
Change in HIV prevalence among MSM at journey destination compared to journey origin in China, 2015

Sex tourists came primarily out of Eastern and Northern regions of China. Sex tourists traveled from an origin with a lower MSM HIV prevalence to a destination with a higher MSM HIV prevalence in 61 out of 92 sex tourism trips. Figure 1 shows the relationship between the MSM HIV prevalence at the origin and the change of prevalence at the destination. Paired sample analysis showed that the MSM HIV prevalence at sex tourism journey origins were lower than MSM HIV prevalence at the destinations (mean = 6.86, SD = 5.24; paired T-test t(91) = − 4.218, *p* < 0.01).

## Discussion

We identified a subset of MSM sex tourists with multiple high-risk sexual behaviors. Most MSM sex tourist research has focused on high-income countries [7, 10, 17] or specifically recruited participants who have traveled recently. [7, 8, 10, 22] Our study extends the literature on sex tourism by including data in a middle-income country, comparing origin and destination HIV prevalence, and comparing the differences between those traveling primarily to purchase sex and those traveling primarily for other reasons.

We found that MSM sex tourists represented a broader group than that recognized by the World Tourism Organization, a subsidiary of the United Nations. This organization defines sex tourism as “trips organized … using [tourism] networks …with the primary purpose of effecting a commercial sexual relationship by the tourist with residents at the destination.” [23] This operational definition has been used in previous research. [10, 22] Our data suggest that in China, this definition is no longer adequate and that many MSM sex tourists will travel for primary reasons other than purchasing commercial sex. In fact, nearly two-thirds of our sex tourists would not be captured using this original definition. Comparison of the primary sex tourists who fall within the original definition and the non-primary sex tourists who do not shows that there is no significant difference in socio-demographic and sexual behavior characteristics, suggesting a broader definition is more appropriate. In addition, both primary and non-primary sex tourists reported their primary motivation to purchase sex while traveling was due to fear of recognition at home as well as a desire to try sex with males. This supports previous findings that Chinese MSM are driven to participate in sex tourism because of the strong social stigmas and bias against MSM. [24] A previous study has also argued for the need to include non-primary sex tourists in sex tourism research, and reported that sexual services are often purchased by those businessmen and others staying long-term at their destination. [9] We further broadened the definition of sex tourism by including domestic journeys as well as international journeys. Had we not defined sex tourism to include domestic travel, 75% of our sex tourists would not have been identified. By using our broader definition of sex tourism, we were able to capture a wider spectrum of individuals engaging in the same risky sexual behaviors. Thus, our data also suggest that previous research likely underestimates the frequency of MSM sex tourism. [10, 22]

Sex tourists displayed several high-risk sexual behaviors such as condomless sex, sex while drunk, group sex, multiple sex partners and having been paid for sex. These findings are consistent with findings from China, [24], the United Kingdom, [7] Belgium, [10] the United States, [8] and Vietnam. [22] This suggests that sex tourism may contribute to the co-occurrence of risk and disease syndemics. [25] Our participants reported slightly higher rates of condom usage at home than when traveling (23% versus 34%, respectively), contrasting existing research showing that sex tourists are more likely to use condoms while traveling. [24] Unprotected sex while traveling, in combination with other correlated risk behaviors such as group sex, sex while using drugs, sex while drunk and multiple sex partners, amplifies disease transmission. [5] This trend is consistent with the higher burden of living with HIV noted among sex tourists in our sample. Efforts to reduce HIV/STI transmission risks while traveling may be effective in reducing disease spread upon return. Delivering information on disease transmission and prevention prior to departure has been shown to reduce STI risk in Dutch marines prior to Cambodian travel, American Peace Corps volunteers in Africa and Swiss airline workers. [9]

Another key finding of our study was that sex tourists tend to travel from locations with low MSM HIV prevalence areas to locations with high MSM HIV prevalence, consistent with previous research from China. [26] This is likely related to the higher known burden of HIV in urban areas, [20] to where domestic sex tourists tended to travel. This presents a concern for disease transmission when travelers return to their home regions with lower HIV prevalence. [6] Previous studies on sex tourism in China [27] and internationally [5] have shown a trend in travel from regions of lower HIV prevalence to higher HIV prevalence, leading to transmission upon return. However, given the large migrant population in China, it may be challenging to isolate disease transmission secondary to migration from sex tourism. Studies targeted towards better understanding disease transmission dynamics are needed to illuminate the impact of sex tourism on disease transmission in China. Furthermore, the lack of access to healthcare and the low retention in the HIV treatment cascade among both the migrant population and sex tourists is extremely concerning. [28]

Some limitations of our study should be noted. First, our participants were recruited for an online survey, and MSM recruited through the Internet in China are on average younger and better educated. [29] In addition, we only recruited high-risk men, and men who consistently used condoms were not captured in this analysis. However, we believe this selection makes our findings even more relevant to HIV transmission. Second, as the survey was a cross-sectional study, it impossible to infer causal relationships or to interpret baseline activity. Questions on sex tourism and sexual risk behaviors were asked without a specific time frame. As we gathered data on MSM HIV prevalence from recent sources, these numbers changed and we cannot confidently correlate that to the MSM HIV prevalence during the time period each individual was traveling. Third, the definitions and indicators used by each source may have varied from both each other and our own definitions. Fourth, the small number of sex tourists limited our ability to examine some associations. However, as our study was a cross-sectional, observational study, the small percentage we noted may be a better estimation of the frequency of sex tourism among MSM in China. Fifth, although our study collected information on the price paid for sexual activities, we did not ask about which sexual activities were purchased. The lacking of this information limited our ability to know the cost of sexual tourism of the participants. Finally, only 62 people indicated that they engaged in sexual tourism. This small size limited our ability to conduct additional sub-analyses.

While it is becoming increasingly clear that sex tourism poses an international public health risk, more studies on the topic are necessary. Targeted studies using phylogenetic research would give a better understanding disease transmission. Site-specific phylogenetic studies have been able to provide definitive evidence of HIV transmission between Singapore and Malaysia, [5] in turn helping to more accurately design and target interventions. Our behavioral data is consistent with phylogenetic research suggesting sexual mixing between Thai and Chinese MSM. [30] Similar studies looking at travel within China would shed more light on the disease transmission due to migration.

Interventions need to be set in place to prevent disease transmission as a result of sex tourism. Using procedures requiring few resources, it may be possible to identify sex tourists for more targeted intervention. Facilitating healthcare access for MSM and implementing policies requiring healthcare providers to ask about recent travels and relevant sexual history may identify cases that would otherwise go unnoticed. In addition, giving pre-departure education to travelers either in health settings such as clinics or travel websites would also better inform travelers. At destinations popular for sex tourism, promoting healthcare access in the form of travel clinics and public advertisements promoting sexual health may also mitigate disease transmission risks. As several of our participants reported smartphone app use to locate a sexual partner, use of smartphones to deliver interventions may be useful in the future to target sex tourists both pre- and post-departure.

## Conclusion

Sex tourism occurs in several countries, and has long been associated with risky sexual behavior, [7–10] presenting significant public health concerns. The stigma associated with MSM behaviors will likely persist in low- and middle-income countries, increasing the need for improved public health programs and research. Given existing syndemics among MSM and differential HIV prevalence in origin and destination locations, sex tourism could amplify regional MSM HIV epidemics.

## Abbreviations

aOR: Adjusted Odds Ratio
cOR: Crude Odds Ratio
HIV: Human Immunodeficiency Virus
IRB: Institutional Review Board
MSM: Men who have sex with men
STI: Sexually Transmitted Disease
